# Unravelling the unseen threat of polyhydramnios in patients with gestational diabetes mellitus: A case report

**DOI:** 10.51866/cr.377

**Published:** 2023-12-21

**Authors:** Syamimi Nadiah A Wahab, Abdul Hadi Said, Wan Hasmawati Wan Ismail

**Affiliations:** 1 MBBS, Department of Family Medicine, International Islamic University Malaysia, Kuantan, Pahang, Malaysia. Email: syanaawe24@gmail.com; 2 MD, MMed, Department of Family Medicine, International Islamic University Malaysia, Kuantan, Pahang, Malaysia.; 3 BMBS, MMed Klinik Kesihatan Paya Besar, Kuantan, Pahang, Malaysia.

**Keywords:** Polyhydramnios, Congenital Diaphragmatic hernia, Edwards syndrome, Gestational diabetes mellitus

## Abstract

Polyhydramnios is defined as an increase in the amount of amniotic fluid during pregnancy. This article presents the case of a 35-year-old G4P3 lady at 28 weeks of gestation with suboptimised gestational diabetes Mellitus (GDM). Routine transabdominal ultrasound showed the presence of polyhydramnios, initially thought to be due to suboptimal glucose control. Further evaluation revealed a congenital diaphragmatic hernia with multiple soft markers. Identifying the underlying causes of polyhydramnios can be challenging in primary care settings, which can be attributed to various factors. Although primary care medical officers may not be required to perform detailed scans, they have a crucial role in identifying gross foetal abnormalities. This study highlights the potential for missed diagnoses in primary care settings and the importance of comprehensive prenatal assessments to ensure early detection and appropriate management of polyhydramnios-related conditions in women with GDM.

## Introduction

Polyhydramnios, characterised by excessive amniotic fluid accumulation during pregnancy, requires thorough investigation and management.^1^ It affects approximately 0.5%–1.5% of pregnancies.^2^ While most cases are idiopathic, congenital anomalies contribute to 21.3% of cases, especially those with moderate-to-severe degrees, with gastrointestinal malformations being the commonest.^1-3^ Among these cases, congenital diaphragmatic hernia (CDH) is a marked anomaly detected in 3.2% of patients.^3^ Additionally, trisomy 18 is the most frequently diagnosed genetic syndrome in patients with polyhydramnios and CDH.^3,4^ This article presents a case of moderate-to-severe polyhydramnios in a pregnant woman with a foetus diagnosed with CDH and underlying Edwards syndrome. Some challenges encountered by primary care professionals in detecting such anomalies relate to professional expertise, scan mode, transducer frequency, small defects in early gestation and late development of the underlying disease.^5-7^ While primary care medical officers may not be required to conduct detailed scans, it is essential for them to recognise major anomalies diligently. This study aims to raise awareness among primary care professionals about comprehensive evaluation and diligent consideration and ruling out of other potential causes of polyhydramnios beyond suboptimal gestational diabetes mellitus (GDM) for timely and accurate management, benefitting maternal and foetal outcomes.

## Case presentation

A 35-year-old G4P3 lady at 28 weeks of gestation came for a routine antenatal checkup. She was diagnosed with GDM at 18 weeks of gestation and was on oral glucose-lowering drugs, as her blood sugar profile monitoring was suboptimised. She had no history of infection, osmotic diuresis or hypoglycaemic symptoms throughout her pregnancy. Her marriage was non-consanguineous, and both her and her husband’s families had no history of congenital anomalies or syndromes. There was also no history of supplement or traditional medication consumption. All her previous pregnancies were uneventful and all her children were healthy.

**Figure 1:**
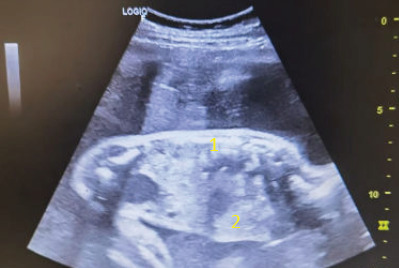
Sagittal view of the foetus. The heart (1) and stomach bubble (2) are seen together in the thoracic cavity.

**Figure 2:**
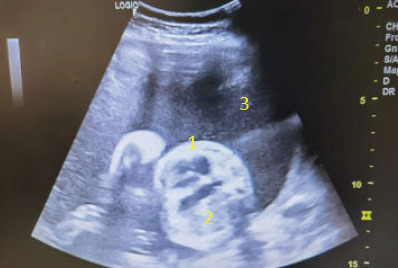
Axial view of the foetus. The heart (1) and stomach bubble (2) are seen together on the same axis. The heart is pushed towards the right side. Polyhydramnios (3) is also present.

On physical examination, the symphysial fundal height was 31 cm and the foetal parts were difficult to palpate. Transabdominal ultrasound revealed polyhydramnios with a deepest vertical pocket (DVP) of 12 cm, suggesting a ‘uterus larger than date’. This finding raised the operator’s suspicion to identify other causes of polyhydramnios apart from suboptimal GDM. Assessment of the growth parameters showed that the foetal abdominal circumference (AC) was much smaller than the other parameters, corresponding to 25 weeks of gestation, while the other parameters corresponded to 28–30 weeks of gestation. Furthermore, a stomach bubble was absent. The stomach bubble was noted in the thoracic cavity (Figure 1) and the heart was pushed to the right side (Figure 2). Other soft markers were found, such as cleft lip and abnormal hands. Her previous antenatal scan was unremarkable. The patient was referred to a maternal–foetal medicine (MFM) consultant to rule out CDH with multiple soft markers.

A detailed scan by an MFM consultant revealed that the foetus had multiple anomalies such as left-side cleft lip, claw hand, rocker bottom feet, stomach bubble in the thoracic cavity with a lung-to-head ratio of 0.58 and polyhydramnios with a DVP of 12 cm which consistent with CDH. Other organs and structures, including the brain, spine, heart, abdominal wall, bladder and kidneys were normal. The estimated foetal weight was 1182 grams. An amniocentesis was conducted and sent for karyotyping confirming a male (XY) genotype affected with trisomy 18 with normal copy numbers of chromosomes 13 and 21, which suggested Edwards syndrome.

After the karyotyping result was reviewed, the parents were informed regarding the diagnosis and prognosis. They were given the option of expectant management or early delivery where the baby will receive comfort care and expectant management in the event of delivery. The possibility of foetal demise, which could happen prenatally, intranatally or postnatally, was also being discussed. During subsequent follow-up, the patient had symptomatic polyhydramnios with an amniotic fluid index (AFI) of 43 cm and an amnioreduction was performed. The patient delivered via spontaneous vertex delivery at 34 weeks of gestation, with the foetus having no signs of life.

## Discussion

Polyhydramnios, characterised by an excessive accumulation of amniotic fluid during pregnancy, is a condition that can present various challenges and implications for both the mother and foetus. It is classified as mild with a DVP of 8–11 cm (AFI of 25–30 cm), moderate with a DVP of 12–15 cm (AFI of 30.1–35 cm) or severe with a DVP above 16 cm (AFI of ≥35.1 cm).^8^ With mild polyhydramnios, an underlying disease is identified in only 17% of cases, while with moderate-to-severe polyhydramnios, an underlying disease is detected in 91%.^8^ In a separate study on patients with moderate-to-severe polyhydramnios, the prevalence of foetal malformations was notably high, reaching almost 80%.^3^

In pregnancies affected by polyhydramnios, approximately 20% of cases are due to congenital anomalies, including gastrointestinal anomalies, central nervous system defects, musculoskeletal anomalies, airway malformations and CDH, while 60%–70% remain idiopathic with no discernible cause.^8^ Other foetal causes include intrapartum infections (i.e. TORCH infections), chromosomal abnormalities and urogenital disorders.^8^ Common maternal causes of polyhydramnios include GDM and alloimmunisation secondary to maternal antibodies resulting in foetal haemolytic anaemia.^1,9^ In the present case, the patient was diagnosed with GDM at 18 weeks of gestation and polyhydramnios during a routine prenatal visit at 28 weeks of gestation. Polyhydramnios was the first suspicious sign the primary care medical officer noticed. Therefore, the presence of polyhydramnios must not deter primary care medical officers from investigating other potential causes apart from GDM alone.

At the primary care level, the approach to polyhydramnios should focus on a comprehensive assessment to identify potential causes and associated anomalies. While primary care medical officers may not be required to conduct detailed scans, they play a vital role in recognising gross foetal abnormalities. In this case, apart from moderate polyhydramnios, certain ultrasound findings such as a small AC, the absence of a stomach bubble and the presence of a stomach bubble in the thoracic cavity, raised the medical officer’s suspicion, as these are considered obvious signs of congenital abnormalities. The quality of scans in primary care clinics can be influenced by ultrasound skill and experience, transducer frequency, limited advanced ultrasound machines, small defects in early gestation and late development of the underlying disease.^5-7^ This limitation could impact the accuracy of detecting complex anomalies, such as CDH. Therefore, advocating for local guidelines, strengthening resources and providing enhanced training in primary care settings can contribute to early detection and appropriate management of polyhydramnios-related conditions.

CDH is a diaphragmatic deformity caused by failed closure of the pleuroperitoneal canal at approximately 10 weeks of gestation, resulting in herniation of abdominal contents into the thoracic cavity and compression of lung tissues.^10^ Approximately 60% of cases are detected prenatally either on routine ultrasound (mean gestational age of 24.2 weeks) or as part of a workup for maternal polyhydramnios.^11^ The mortality rate of CDH continues to be high, ranging from 20% to 60%. It is particularly high when the condition is associated with a chromosome abnormality, and most affected babies do not survive beyond the first few weeks or months of life.^12^ The most common abnormality is trisomy 18, which affects 10% of all individuals with CDH.^4^ In our case, the foetus was diagnosed with CDH and underlying trisomy 18 at 28 weeks of gestation, resulting in a fresh stillbirth delivery at 34 weeks of gestation.

Early detection is important for the benefit of both parents and the baby, as it allows for timely and informed decision-making, access to specialised care and interventions and the opportunity to plan for appropriate medical management and support.^11^ This goal can be achieved in multiple ways such as comprehensive prenatal ultrasound screening, especially during the second trimester; training and education of primary care medical officers in recognising common ultrasound findings associated with foetal anomalies; and genetic counselling and screening, especially for those at a higher risk of chromosomal abnormalities. However, CDH can be missed during early pregnancy owing to the smaller diaphragmatic defect and late development of CDH in a substantial proportion of patients.^13^

Early detection and prenatal diagnosis enable parental counselling, referral to a tertiary care centre and intervention for high-risk foetuses.^11^ Detailed ultrasound, foetal karyotyping and microarray analysis are needed to confirm the diagnosis and to identify related abnormalities and chromosomal abnormalities, as in our case, which is consistent with Edwards syndrome. After discussing the prognosis, they decided to opt for expectant management and comfort care, as the survival rate was low. Parents should be informed about this beforehand so they can psychologically and emotionally prepare for any circumstance that may arise. Any parents who find it difficult to handle their emotional stress can be referred to a counsellor or clinical psychologist. Counselling and support play a pivotal role in helping parents navigate their emotions and empower them to make informed decisions. Primary care professionals should be aware of the significance of counselling to guide and support parents effectively because parents who are unaware of their child’s well-being during pregnancy are bound to be taken aback by an unexpected stillbirth or infant death.

## Conclusion

This case report emphasises the significance of considering alternative differential diagnoses when encountering polyhydramnios in patients with GDM, as other more severe diseases may be the cause. Therefore, it is imperative that patients at a high risk of congenital abnormalities undergo a thorough assessment by an experienced primary care physician especially when routine ultrasound findings are abnormal. The present findings may be used as a basis to educate primary healthcare practitioners on managing polyhydramnios while focusing on CDH as one of the devastating causes of polyhydramnios that must not be missed. Early detection and referral to other multidisciplinary teams can guide the management and prognosis and prepare parents for any circumstances that may arise.
